# Trends in prostate cancer incidence and mortality in Croatia, 1988 to 2008

**DOI:** 10.3325/cmj.2012.53.109

**Published:** 2012-04

**Authors:** Tomislav Kuliš, Ivan Krhen, Željko Kaštelan, Ariana Znaor

**Affiliations:** 1 Department of Urology, University Hospital Center Zagreb, University of Zagreb School of Medicine, Zagreb, Croatia; 2 Croatian National Cancer Registry, Croatian National Institute of Public Health, Zagreb, Croatia; 3 Andrija Štampar School of Public Health, University of Zagreb School of Medicine, Zagreb, Croatia

## Abstract

**Aim:**

To describe and interpret prostate cancer incidence and mortality trends in Croatia between 1988 and 2008.

**Methods:**

Incidence data for the period 1988-2008 were obtained from the Croatian National Cancer Registry. The number of prostate cancer deaths was obtained from the World Health Organization mortality database. We also used population estimates for Croatia from the Population Division of the Department of Economic and Social Affairs of the United Nations. Age standardized incidence and mortality rates were calculated by the direct standardization method. To describe time trends of incidence and mortality, joinpoint regression analysis was used.

**Results:**

Average age-standardized incidence rate between the first and last five-year period doubled, from 19.0/100 000 in 1988-1992 to 39.1 per 100 000 in 2004-2008. Age-standardized mortality rate increased by 6.9%, from 14.5 to 15.5 per 100 000. Joinpoint analysis of incidence identified two joinpoints. The increasing incidence trend started from 1997, with the estimated annual percent of change (EAPC) of 12.9% from 1997-2002 and of 4.1% from 2002-2008. Joinpoint analyses of mortality identified one joinpoint. Mortality trend first decreased, with EAPC of -3.0% from 1988-1995 to increase later with EAPC of 2.0% from 1995-2008.

**Conclusion:**

The incidence of prostate cancer in Croatia has been on the increase since 1997. Trend in mortality is increasing, contrary to the trends in some higher-income countries. An improvement in the availability of different treatment modalities as well as establishing prostate cancer units could have a positive impact on prostate cancer mortality in Croatia.

Prostate cancer has become the most common male cancer in Western populations and the third most common cause of cancer death in Europe (1). In Croatia, it is the third most common male cancer after lung and colorectal cancer. In 2008, 1692 men were diagnosed with prostate cancer and 641 men had prostate cancer certified as cause of death (2,3).

There are three well-established risk factors for prostate cancer: increasing age, ethnic origin, and heredity (4). Other factors have also been discussed, such as nutrition, pattern of sexual behavior, alcohol consumption, exposure to UV radiation, and occupational exposure (5).

So far, primary prevention of prostate cancer has not been possible, but there are means for secondary prevention. Prostate-specific antigen (PSA) testing was introduced more than 20 years ago (6) and ever since has had a great impact on early prostate cancer detection. However, the existing evidence from meta-analyses of randomized controlled trials does not support the routine use of screening for prostate cancer with PSA (7). In Croatia, PSA testing is applied as a mode of opportunistic screening, defined as individual case findings, which are initiated by the patient and/or his physician.

Prostate cancer presents significant burden for society and with the aging of population its incidence is expected to rise further. The aim of this study is to describe and interpret prostate cancer incidence and mortality trends in Croatia between 1988 and 2008 and to compare the current trends to other European countries and propose potential changes in health service to further enhance prostate cancer management.

## Materials and methods

### Data sources

Incidence data were obtained from the Croatian National Cancer Registry. The Registry, founded in 1959, covers the whole Croatian population (approximately 4.4 million persons) and relies on mandatory cancer notifications from primary and secondary health care sources and death certificates from the Croatian Bureau of Statistics. The Registry has contributed data to the last three volumes of the Cancer Incidence in Five Continents series (8-10). Prostate cancer was defined as ICD-10 C61 and ICD-9 185. The number of prostate cancer deaths was obtained from the WHO mortality database (11). For calculating age-specific rates for 5-year age groups, we used the population estimates from the Population Division of the Department of Economic and Social Affairs of the United Nations (12).

### Statistical analysis

Age-standardized rates of cancer incidence in Croatia and truncated age standardized rates (at ages 35-64) were calculated by the direct standardization method, using the world standard population as a reference (13). To describe incidence and mortality time trends, we carried out joinpoint regression analysis using the software Joinpoint Regression Program, Version 3.5.2. October 2011. The analysis included logarithmic transformation of the rates, standard error, maximum number of five joinpoints, and minimum of four years between two joinpoints. All other program parameters were set to default values. The aim of the approach is to identify possible joinpoints where a significant change in the trend occurs. The method identifies joinpoints based on regression models with 0-5 joinpoints. The final model selected was the most parsimonious of these, with the estimated annual percent change (EAPC) based on the trend within each segment (14). To quantify the trend over a fixed number of years, the average annual percent change (AAPC) was calculated. The AAPC is computed as a geometric weighted average of the EAPC trend analysis, with the weights equal to the lengths of each segment during the prespecified fixed interval (15).

In describing trends, the terms “significant increase” or “significant decrease” signify that the slope of the trend was statistically significant (*P* < 0.05). For non-statistically significant trends (*P* > 0.05), we used the terms “stable” (for EAPC between -0.5% and 0.5%), “non-statistically significant increase” (for EAPC>0.5%), and “non-statistically significant decrease” (for EAPC<-0.5%). All statistical tests were two sided.

## Results

**Figure 1:**
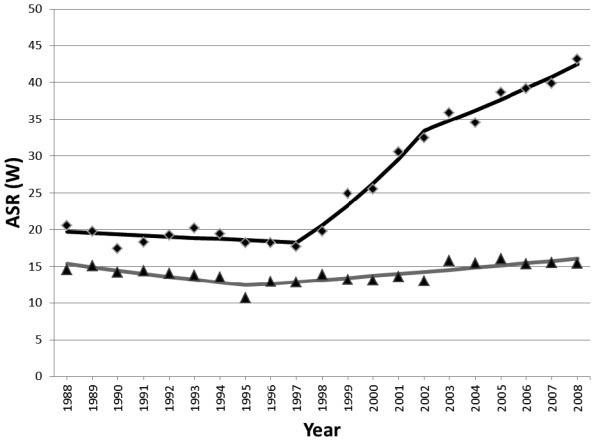
Joinpoint analysis for incidence and mortality of prostate cancer in Croatia, 1988-2008. Rhomb – incidence; triangle – mortality; ASR (W) – age-standardized rate (world standard population).

Age-standardized incidence rate between the first and last five years increased by 105.8%, from 19.0 to 39.1 per 100 000, while age standardized mortality rate increased by just 6.9%, from 14.5 to 15.5 per 100 000 (Table 1). Incidence trend exhibited significant increase (Figure 1). Joinpoint analysis identified two joinpoints, in 1997 and 2002, with consequent three trends (Table 2). The period 1988-1997 was characterized by a non-significant decrease, with EAPC of -0.8% (95% confidence interval [CI], -2.4% to 0.8%). The second and third period showed significantly increasing trends, with EAPC of 12.9% (95% CI, 8.0% to 18%) and 4.1% (95% CI, 2.2% to 6%), respectively. The trend since 1997 was significantly increasing, with AAPC 8.0% (95% CI, 5.8% to 10.2%).

**Table 1:** Prostate cancer incidence and mortality data in the period of 1988 to 2008. Number of cases, crude rate, and age standardized rate per 100 000

Year	Incidence			Mortality		
	N	crude rate	ASR*	N	crude rate	ASR
**1988**	558	25.7	20.6	407	18.7	14.6
**1989**	533	24.5	19.7	413	19.0	15.1
**1990**	492	22.5	17.4	398	18.2	14.2
**1991**	513	23.3	18.3	411	18.7	14.4
**1992**	560	25.3	19.2	410	18.5	14.0
**1993**	610	27.3	20.2	417	18.7	13.8
**1994**	615	27.4	19.4	426	19.0	13.5
**1995.**	631	28.0	18.2	354	15.7	10.7
**1996**	618	27.6	18.2	432	19.3	12.9
**1997**	613	27.5	17.7	441	19.8	12.8
**1998**	688	31.2	19.8	474	21.5	13.9
**1999**	882	40.3	25.0	463	21.2	13.2
**2000**	923	42.5	25.5	466	21.5	13.1
**2001**	1110	51.4	30.5	478	22.1	13.6
**2002**	1211	56.3	32.4	488	22.7	13.0
**2003**	1357	63.2	35.9	601	28.0	15.8
**2004**	1316	61.4	34.5	591	27.6	15.4
**2005**	1498	70.1	38.7	636	29.7	16.0
**2006**	1515	71.0	39.2	604	28.3	15.4
**2007**	1580	74.2	39.9	637	29.9	15.5
**2008**	1692	79.6	43.2	641	30.1	15.4

*ASR (W) – age-standardized rate per 100 000 (using world standard population).

**Table 2.** Joinpoint analyses of incidence and mortality of prostate cancer with the estimated annual percent change (EAPC) and 95% confidence interval (CI)

Joinpoint analyses of mortality identified one joinpoint at the year 1995, with two trends (Table 2). In the first period, the mortality trend showed a non-significant decrease, with EAPC of -3.0% (95% CI, -4.9% to -1.1%), while in the second period it significantly increased, with EAPC of 2.0% (95% CI, 1.3% to 2.7%). The overall trend was stable, with AAPC of 0.2% (95% CI, -0.5% to 1%).

Joinpoint analyses for mortality were also performed on age standardized mortality rates truncated to male population 35-64 years old. In this population, the trend showed a non-significant decrease, with EAPC of -0.6% (95% CI, -1.8% to 0.6%).

## Discussion

Compared to GLOBOCAN 2008 estimates for other European countries, Croatia has an intermediate incidence rate of prostate cancer (16). With age-standardized rate (ASR) of 43.2/100 000, it ranked 25th of 40 European countries. The highest ASR, of 126.3/100 000, was found in Ireland and the lowest, of 12.5/100 000, in Moldova. According to mortality, Croatia ranked 9th, with ASR of 15.4/100 000. The highest rate, of 22.0/100 000 was found in Estonia and the lowest, of 6.6/100 000, in Moldova.

All European countries and the US have experienced an increase in prostate cancer incidence (17,18). The most prominent increase in the US occurred in the early 1990s, with a peak in 1992 as a result of a larger-scale PSA testing (18). Similar increases are described in some other European countries with the change of trend in the following years: Denmark in 1995, EAPC 7.2%; Finland in 1990, EAPC 9.3%; Latvia in 1994, EAPC 11.0%; Lithuania in 1991, EAPC 8.1%; Norway in 1988, EAPC 4.0%; Sweden in 1996, EAPC 6.9%; and the Czech Republic in 1990, EAPC 6.9% (17). In Croatia, prostate cancer incidence was stable from 1988 to 1997. The increase was noted in the period after 1997, when the incidence increased, with EAPC of 12.9%. This increase could be attributed to a wider acceptance of PSA testing (19), improved prostate biopsy techniques (20), and increased awareness of prostate cancer in Croatian male population.

Prostate cancer has become the most common non-skin cancer in European men (1). The increase in its incidence was influenced by increased public awareness, PSA testing, and higher detection of latent cancer in prostate surgery (21-24). Prostate cancer is very common in older men and autopsy data show that more than 50% of men older than 70 years have indolent prostate cancer (25-27). Furthermore, it is characterized by slow growth and there is a common saying that most men die with the disease, not from it. However, prostate cancer patients have a higher risk of dying from various other causes (28). Even for men with aggressive disease there is a time lag between diagnosis and death. Currently, we cannot predict which lesion will progress and which will stay indolent (29). Since PSA testing has a considerable effect on increase in prostate cancer incidence (30), there is a large debate over potential overdiagnosis of prostate cancer (18,31). The European Randomized Study of Screening for Prostate Cancer during a median follow-up of nine years reported that PSA-based screening reduced the rate of death from prostate cancer by 20% (32). Yet, this came with a considerable risk of overdiagnosis (defined as the diagnosis in men who would not have clinical symptoms during their lifetime), meaning that 1410 men would need to be screened and 48 additional cases would need to be treated to prevent one death from prostate cancer. Furthermore, for one man to experience a presumed benefit, more than 20 would have to be diagnosed (18). Additionally, Bray et al reported that in the recent years in some European countries the correlation between incidence and mortality has disappeared, which is consistent with the overdiagnosis or detection of indolent tumors, most likely attributable to PSA testing. The highest incidence rates are reported in the countries with high health care expenditure, which also supports this notion (17).

Mortality trend in the overall period appears stable, however, joinpoint analysis identified a significant increase in mortality since 1995, with EAPC of 2%. Increase in mortality could be attributed to a potential role of increased diagnosis and certification of latent prostate cancer in older age patients – mainly following the increase in PSA testing. To exclude this, we performed joinpoint analyses of age-standardized mortality rates truncated to the population 35-64 years old. Here, joinpoint identified a non-significant decrease, with EAPC of -0.6%.

However, in 15 of 24 European countries, there was a decrease in mortality despite the increase in incidence (33). It remains unclear whether the observed decreases in mortality are associated with advancement in treatment and/or wider level of PSA testing (17,18,34). It is also unclear to what extent the increases in mortality in other countries are the result of an increased detection of latent cases or of a true change in risk. However, it must be noted that the decreases in mortality are mostly present in higher income countries, ie, countries with high health care expenditures (eg, Germany, the UK, France, Norway) (33).

Clinical management of prostate cancer has advanced significantly over the last two decades. Earlier cancer diagnosis, as a result of wider use of opportunistic PSA testing, has resulted in more frequent surgical treatment (ie, radical prostatectomy), especially in younger men. New treatment modalities have emerged, such as brachytherapy, hormonal therapy, and modern radiotherapy techniques (eg, three-dimensional conformal radiotherapy and intensity-modulated radiotherapy) (35). For locally advanced disease, combination of different modality treatments with long term hormonal therapy, as well as a second-line treatment in case of recurrence, has become an accepted mode of treatment. Availability and accessibility of different modern treatment modalities has definitely had a significant influence on mortality decline.

Treatment of prostate cancer requires multidisciplinary approach. Therefore, in Europe there is an incentive to organize prostate cancer units (36). These units would be responsible for diagnosis, staging, and management of prostate cancer patients. It is expected that they will provide holistic, multiprofessional management of the disease and potentially avoid inappropriate treatments and secondary therapies (36).

The incidence of prostate cancer in Croatia has been on a continuous increase since 1997 and has not plateaued so far. The mortality trend is increasing, contrary to the trends in some higher-income countries. It remains unclear to what extent the increase in mortality is a result of improved diagnosis and certification of prostate cancer. However, improving the availability of different treatment modalities and establishing prostate cancer units could have a positive impact measured as a decrease in mortality.

## 

**Table:** 

	Trend 1			Trend 2			Trend 3		
	years	EAPC*	95% CI	years	EAPC	95% CI	years	EAPC	95% CI
Incidence	1988-1997	-0.8	-2.4 to 0.8	1997-2002	12.9*	8.9 to 18.0	2002-2008	4.1*	2.2 to 6.0
Mortality	1988-1995	-3.0*	-4.9 to -1.1	1995-2008	2.0*	1.3 to 2.7			

*Significant EAPC.
